# Supplementary material for: Amphiphilic Silver Nanoparticles for Inkjet-Printable Conductive Inks
Source: Nanomaterials (Basel). 2022 Nov 29;12(23):4252. doi: 10.3390/nano12234252 (PMC9739383; doi:10.3390/nano12234252)
Supplement: Supplementary file 1 [file nanomaterials-12-04252-s001.zip › nanomaterials-2035925-supplementary.pdf]

nanomaterials

Supplementary Materials

Amphiphilic Silver Nanoparticles for Inkjet-Printable Conductive Inks

Irena Ivanišević [1], Marin Kovačić [1], Marko Zubak [1], Antonia Ressler [1,2], Sara Krivačić [1], Zvonimir Katančić [1], Iva Gudan Pavlović [1] and Petar Kassal [1,*]

1 Faculty of Chemical Engineering and Technology, University of Zagreb, Marulićev trg 19, 10000 Zagreb, Croatia
2 Faculty of Engineering and Natural Sciences, Tampere University, Korkeakoulunkatu 6 P.O. Box 589, 33014 Tampere, Finland
* Correspondence: pkassal@fkit.hr

Table S1. Cartesian coordinates of the optimized Ag_8 cluster:.

**Atom no.**	Atom type	Coordinates (Å)		
		X	Y	Z
**1**	Ag	-1.330863	-1.015962	0.786259
2	Ag	-4.052076	-1.285159	0.126792
3	Ag	-3.127554	0.33548	2.319152
4	Ag	-1.617338	1.732624	0.305806
5	Ag	-2.069716	-2.284672	-1.566161
6	Ag	-2.875574	0.374631	-1.767217
7	Ag	-0.121895	0.139711	-1.442053
8	Ag	-4.455175	1.574894	0.127593

Table S2. Cartesian coordinates of the optimized PAA-NMP system:.

**Atom no.**	Atom type	Coordinates (Å)		
		X	Y	Z
**1**	C	-0.974293	1.242726	0.010649
2	H	-1.464108	1.281135	-0.973776
3	C	-2.020891	1.466518	1.079126
4	O	-1.774677	1.674125	2.242357
5	O	-3.265252	1.38113	0.60777
6	H	-3.874603	1.511994	1.353808
7	C	0.123755	2.305355	0.093968
8	H	0.390672	2.456128	1.151881
9	H	1.021002	1.92225	-0.414229
10	C	-0.242732	3.646943	-0.547912
11	H	-0.450012	3.468531	-1.613724
12	C	0.953971	4.57527	-0.515635
13	O	1.535104	4.944692	-1.515569
14	O	1.270025	4.955821	0.704669
15	H	1.888816	5.754468	0.678448
16	C	-1.452669	4.315259	0.11512
17	H	-2.357273	3.733539	-0.116835
18	H	-1.320573	4.278259	1.207456
19	C	-1.689025	5.770336	-0.298643
20	H	-0.828695	6.381843	0.009685

21	C	-2.85321	6.374457	0.447002
22	O	-2.871229	7.506084	0.871286
23	O	-3.893982	5.547968	0.570708
24	H	-4.598713	6.027374	1.036868
25	C	-1.847731	5.931651	-1.820495
26	H	-0.873785	5.693548	-2.269406
27	H	-2.565085	5.186371	-2.200375
28	C	-2.291023	7.31002	-2.326115
29	C	-1.378169	8.408264	-1.825328
30	O	-0.212859	8.251817	-1.543547
31	O	-1.965664	9.601986	-1.779912
32	H	-1.297118	10.253829	-1.514129
33	H	-2.075266	7.310907	-3.410925
34	C	-0.395151	-0.170881	0.185802
35	C	-1.455346	-1.26387	0.356013
36	H	0.257994	-0.182465	1.073842
37	C	0.464026	-0.522856	-1.010705
38	H	-2.162413	-1.262063	-0.486948
39	H	-2.02396	-1.11536	1.28482
40	H	-0.980069	-2.252629	0.410914
41	O	0.379187	-0.018461	-2.103587
42	O	1.317314	-1.50658	-0.730733
43	H	1.800112	-1.723319	-1.545195
44	C	-3.795604	7.597322	-2.165302
45	H	-4.293233	6.688086	-1.797077
46	C	-4.517814	7.988666	-3.46741
47	H	-3.970419	8.37268	-1.407413
48	C	-3.855086	9.213432	-4.055827
49	O	-2.910901	9.177012	-4.808404
50	O	-4.38261	10.355242	-3.615623
51	H	-3.872739	11.084552	-4.004077
52	C	-6.017607	8.13184	-3.217443
53	H	-6.346112	7.240512	-2.661408
54	C	-6.88415	8.23091	-4.476344
55	H	-6.205103	8.995876	-2.561512
56	H	-7.940932	8.144122	-4.184687
57	C	-6.712323	9.519164	-5.232159
58	O	-6.03255	9.658	-6.221267
59	O	-7.392164	10.524696	-4.678796
60	H	-7.19721	11.326617	-5.190454
61	H	-4.344635	7.186169	-4.203073
62	H	-6.645892	7.40912	-5.165647
63	C	2.582402	10.022416	-1.283502
64	C	2.64416	8.015066	-0.094818
65	C	3.222025	7.713257	-1.45291
66	C	3.535939	9.089195	-2.032686
67	H	1.62373	10.151315	-1.816007
68	H	3.009523	11.019264	-1.094899
69	H	4.082675	7.035685	-1.370768
70	H	2.44014	7.171948	-2.00816
71	H	4.577533	9.36526	-1.809029
72	H	3.401097	9.145831	-3.120654
73	N	2.347349	9.32251	-0.035345
74	C	1.595082	9.910083	1.035999
75	H	2.076183	10.834515	1.38984
76	H	0.570093	10.148209	0.705546
77	H	1.543168	9.186868	1.859206

78	O	2.439643	7.213534	0.824147

Table S3. Cartesian coordinates of the optimized PAA/Ag_8 system:.

**Atom no.**	Atom type	Coordinates (Å)		
		X	Y	Z
**1**	Ag	-1.330863	-1.015962	0.786259
2	Ag	-4.052076	-1.285159	0.126792
3	Ag	-3.127554	0.33548	2.319152
4	Ag	-1.617338	1.732624	0.305806
5	Ag	-2.069716	-2.284672	-1.566161
6	Ag	-2.875574	0.374631	-1.767217
7	Ag	-0.121895	0.139711	-1.442053
8	Ag	-4.455175	1.574894	0.127593
9	C	6.559234	1.922268	-1.718333
10	C	4.022201	1.544196	-1.658617
11	C	2.622497	2.939727	-0.045919
12	C	2.624097	1.179902	1.799673
13	C	3.321841	-1.067631	2.770976
14	C	3.727892	-2.810616	0.870724
15	C	2.054048	-3.447464	-0.994536
16	H	6.460574	2.958989	-1.364079
17	H	4.223857	0.950284	-0.747538
18	H	1.774432	2.291468	-0.314019
19	H	2.485884	0.474494	0.954967
20	H	2.281201	-1.324245	2.514652
21	H	3.360568	-3.571533	1.578222
22	H	2.855129	-3.837475	-1.642539
23	C	6.742062	1.044323	-0.500432
24	C	2.902182	0.764636	-2.324331
25	C	2.038094	4.319382	0.121021
26	C	1.20811	1.46388	2.246445
27	C	3.589409	-1.75264	4.088901
28	C	4.898286	-3.427288	0.138533
29	C	0.942863	-2.923138	-1.892085
30	O	6.640429	-0.159495	-0.498576
31	O	1.790293	1.243175	-2.516201
32	O	2.433802	5.169436	0.881093
33	O	0.617828	2.502512	2.040464
34	O	3.948589	-1.211136	5.105665
35	O	5.136508	-3.314266	-1.039393
36	O	1.15108	-1.847844	-2.545476
37	O	7.043423	1.741392	0.597332
38	O	3.21591	-0.467845	-2.605686
39	O	1.026683	4.535391	-0.72708
40	O	0.643543	0.425602	2.852648
41	O	3.386447	-3.071041	4.013199
42	O	5.693336	-4.112725	0.965151
43	O	-0.130495	-3.556414	-1.939182
44	H	7.173941	1.109521	1.324548
45	H	2.358114	-1.077493	-2.660643
46	H	0.727797	5.45056	-0.60238
47	H	-0.333026	0.546781	2.852002
48	H	3.598018	-3.454665	4.879819
49	H	6.444212	-4.441077	0.44443
50	C	7.810719	1.824708	-2.59204
51	C	5.309482	1.498514	-2.487896

52	C	3.578525	2.94555	-1.252622
53	C	3.285023	2.436843	1.244986
54	C	3.428536	0.452427	2.89099
55	C	4.264871	-1.617169	1.681566
56	C	2.628488	-2.365658	-0.085833
57	H	7.96386	0.789838	-2.933398
58	H	5.205071	2.14108	-3.377275
59	H	4.457754	3.558215	-0.999254
60	H	4.347216	2.214129	1.062121
61	H	4.487693	0.745513	2.813681
62	H	4.494417	-0.810669	0.967353
63	H	1.807742	-1.919192	0.501942
64	H	8.708654	2.146429	-2.044621
65	H	5.45533	0.467768	-2.842691
66	H	3.086552	3.42426	-2.112806
67	H	3.27352	3.236438	2.000608
68	H	3.101579	0.771639	3.890363
69	H	5.231049	-1.879612	2.139715
70	H	3.041076	-1.564229	-0.710951
71	H	1.655	-4.289791	-0.411271
72	H	7.701907	2.467397	-3.477733

Table S4. Cartesian coordinates of the optimized PAA/Ag_8 system:.

**Atom no.**	Atom type	Coordinates (Å)		
		X	Y	Z
**1**	Ag	-2.759937	-2.685155	-0.593252
2	Ag	-5.088479	-4.21989	-0.142952
3	Ag	-2.649402	-5.451481	-0.493523
4	Ag	-2.895072	-3.99153	1.912664
5	Ag	-5.353506	-1.514282	-0.199762
6	Ag	-5.386568	-2.861635	2.320794
7	Ag	-3.170573	-1.23627	1.821418
8	Ag	-5.239636	-5.634102	2.20268
9	C	-14.525366	3.403875	-1.116526
10	C	-11.948988	3.207556	-1.433786
11	C	-9.517297	2.599639	-2.106203
12	C	-8.255327	3.848995	-0.152876
13	C	-5.731404	4.442362	-0.402326
14	C	-4.288827	3.089169	-2.09005
15	C	-2.322685	1.611681	-1.409229
16	H	-14.192562	4.26113	-0.506502
17	H	-11.851998	3.822871	-0.528102
18	H	-9.798537	2.842517	-3.137353
19	H	-7.834672	2.858964	0.066453
20	H	-5.347199	3.822999	0.42102
21	H	-3.577566	3.927317	-2.170999
22	H	-2.217524	1.370153	-2.475881
23	C	-15.117921	3.931876	-2.422396
24	C	-11.839785	4.135098	-2.634386
25	C	-8.493936	1.477204	-2.154453
26	C	-9.345346	4.181002	0.862795
27	C	-4.763491	5.619894	-0.478863
28	C	-4.471216	2.43803	-3.458607
29	C	-1.996004	0.366908	-0.593283
30	O	-15.898519	3.263261	-3.090256
31	O	-11.916352	3.727564	-3.788864

32	O	-8.289224	0.746146	-1.186325
33	O	-10.26162	4.956646	0.589977
34	O	-3.768519	5.692679	0.227571
35	O	-4.744501	1.243376	-3.563649
36	O	-2.076444	0.480188	0.657952
37	O	-1.731259	-0.686092	-1.227635
38	C	-15.605229	2.650795	-0.350421
39	C	-13.306441	2.501088	-1.390815
40	C	-10.810479	2.164185	-1.423822
41	C	-8.835465	3.865418	-1.563759
42	C	-7.145742	4.913157	-0.015991
43	C	-5.671087	3.621989	-1.690907
44	C	-3.741147	2.078577	-1.093399
45	H	-15.967612	1.801683	-0.947791
46	H	-13.2516	1.747022	-0.58897
47	H	-10.622194	1.865189	-0.380822
48	H	-8.044478	4.141828	-2.270356
49	H	-7.451092	5.787606	-0.614284
50	H	-6.332853	2.751158	-1.589308
51	H	-3.753028	2.509218	-0.082283
52	H	-16.46737	3.298005	-0.129811
53	H	-13.457264	1.942579	-2.329977
54	H	-11.159227	1.254648	-1.939627
55	H	-9.547422	4.694221	-1.594183
56	H	-7.093037	5.280282	1.021487
57	H	-6.073476	4.215428	-2.527131
58	H	-4.421809	1.209654	-1.079267
59	H	-1.605329	2.413675	-1.167479
60	H	-15.208619	2.268753	0.601705
61	N	-4.384026	3.221456	-4.556301
62	C	-4.23633	4.655904	-4.66787
63	H	-4.541889	2.717038	-5.424803
64	H	-4.074259	5.081233	-3.668574
65	H	-3.336185	4.893603	-5.256907
66	C	-5.447304	5.311217	-5.32037
67	H	-6.353602	5.121132	-4.722487
68	H	-5.638998	4.843474	-6.299543
69	C	-5.286646	6.814675	-5.484754
70	H	-6.217446	7.237791	-5.913308
71	H	-5.161161	7.282707	-4.496731
72	N	-4.126284	7.172217	-6.275817
73	C	-4.183065	6.938914	-7.663209
74	C	-3.432353	8.351011	-5.959557
75	C	-3.393398	7.590875	-8.523153
76	H	-4.919083	6.218764	-8.026022
77	C	-2.65122	8.977493	-6.845768
78	H	-3.56691	8.756564	-4.955016
79	H	-3.420656	7.427744	-9.600103
80	O	-2.45231	8.523538	-8.1378
81	H	-2.099234	9.887049	-6.614885
82	N	-5.076338	6.546877	-1.417056
83	C	-4.353492	7.777997	-1.625665
84	H	-5.94943	6.43825	-1.918977
85	H	-3.489202	7.76045	-0.947241
86	H	-3.957877	7.814142	-2.654373
87	C	-5.227885	8.998783	-1.374055
88	H	-5.428786	9.098294	-0.295675

89	H	-6.213466	8.850184	-1.847506
90	C	-4.61305	10.29368	-1.878236
91	H	-5.215246	11.154427	-1.524736
92	H	-3.6066	10.414258	-1.447872
93	N	-4.465615	10.321565	-3.328543
94	C	-5.661598	10.385154	-4.078803
95	C	-3.488658	11.201675	-3.839794
96	C	-5.764533	11.050618	-5.232548
97	H	-6.52881	9.851618	-3.689363
98	C	-3.624909	11.850844	-4.999955
99	H	-2.574173	11.312711	-3.255523
100	H	-6.681244	11.067151	-5.821326
101	O	-4.734519	11.754829	-5.809014
102	H	-2.849591	12.492253	-5.416848
103	N	-9.226854	3.634111	2.088823
104	C	-8.184195	2.729113	2.528651
105	H	-9.954399	3.894683	2.746178
106	H	-7.209594	3.112942	2.186981
107	H	-8.145621	2.779571	3.625009
108	C	-8.365425	1.285243	2.055207
109	H	-8.854158	1.272057	1.069605
110	H	-9.02554	0.733286	2.744063
111	C	-7.044651	0.561026	1.878128
112	H	-7.214461	-0.422762	1.399551
113	H	-6.410282	1.123695	1.1751
114	N	-6.292545	0.379925	3.109064
115	C	-6.555578	-0.783261	3.847358
116	C	-4.952541	0.767481	3.163856
117	C	-5.661293	-1.30225	4.709642
118	H	-7.535096	-1.247387	3.724828
119	C	-4.083481	0.235953	4.041597
120	H	-4.624842	1.541467	2.469131
121	H	-5.86497	-2.179801	5.321985
122	O	-4.448019	-0.71869	4.973941
123	H	-3.053326	0.572871	4.146328
124	N	-7.765147	1.378433	-3.286468
125	C	-7.906278	2.196201	-4.471169
126	H	-6.959414	0.757703	-3.246522
127	H	-8.048	3.251944	-4.191941
128	H	-6.943215	2.167187	-4.995532
129	C	-9.031455	1.762153	-5.411144
130	H	-9.892507	1.399934	-4.827932
131	H	-8.699786	0.905036	-6.019276
132	C	-9.518547	2.913145	-6.280587
133	H	-10.222029	2.537524	-7.050523
134	H	-10.09164	3.610158	-5.649087
135	N	-8.426863	3.666352	-6.867761
136	C	-7.577169	3.027183	-7.78483
137	C	-8.622858	5.019068	-7.16467
138	C	-6.802291	3.713554	-8.630356
139	H	-7.578421	1.937206	-7.801131
140	C	-7.835597	5.684727	-8.016869
141	H	-9.463238	5.520036	-6.683257
142	H	-6.132993	3.247576	-9.351838
143	O	-6.75538	5.095826	-8.653196
144	H	-7.953214	6.74099	-8.254281
145	N	-11.651144	5.44198	-2.323119

146	C	-11.332488	6.448179	-3.314432
147	H	-11.421765	5.640674	-1.351011
148	H	-11.904128	6.228412	-4.228287
149	H	-11.676098	7.424241	-2.942062
150	C	-9.841131	6.516332	-3.629649
151	H	-9.526179	5.595535	-4.144883
152	H	-9.268306	6.537544	-2.689412
153	C	-9.451636	7.701778	-4.492322
154	H	-8.375335	7.625243	-4.754721
155	H	-10.012199	7.670995	-5.438396
156	N	-9.753504	8.981965	-3.870348
157	C	-9.147725	9.270342	-2.631671
158	C	-9.747465	10.094804	-4.733787
159	C	-8.752807	10.500168	-2.282459
160	H	-9.038166	8.452378	-1.919695
161	C	-9.334763	11.309015	-4.356129
162	H	-10.122938	9.920129	-5.743919
163	H	-8.326712	10.731266	-1.306709
164	O	-8.869019	11.603858	-3.090879
165	H	-9.366133	12.180818	-5.00788
166	N	-14.721671	5.168252	-2.785255
167	C	-15.057272	5.780561	-4.052093
168	H	-14.005322	5.614662	-2.225741
169	H	-16.145255	5.961794	-4.100938
170	H	-14.571319	6.767784	-4.055973
171	C	-14.617211	4.989853	-5.280818
172	H	-15.29941	4.14044	-5.430167
173	H	-13.6154	4.568294	-5.102565
174	C	-14.586815	5.854052	-6.531648
175	H	-14.525163	5.213722	-7.427493
176	H	-15.527392	6.421008	-6.630464
177	N	-13.480498	6.797996	-6.544308
178	C	-12.316734	6.464512	-7.247514
179	C	-13.716194	8.178397	-6.505861
180	C	-11.407208	7.380707	-7.60094
181	H	-12.186062	5.420503	-7.535788
182	C	-12.784089	9.072753	-6.850801
183	H	-14.707844	8.507947	-6.193811
184	H	-10.51721	7.145889	-8.182777
185	O	-11.504852	8.715431	-7.250364
186	H	-12.943444	10.149526	-6.829191

Table S5. Complexation energies of the model silver cluster and PAA or MPA-PAA in NMP.

**Energies:**	**Interacting system**	
	**PAA/Ag_8**	**MPA-PAA/Ag_8**
$E_{complex}$, Ha	-3043.796	-5332.899
E_{Ag8}, Ha	-1175.890	-1175.888
E_{PAA}, Ha	-1867.836	
$E_{MPA\text{-}PAA}$, Ha		-4156.932
E_{BSSE}, Ha	0.0245	0.0283
$E_{complexation}$, Ha	-0.0445	-0.0505
$E_{complexation}$, kJ mol^{-1}	-116.76	-132.72

Table S6. Electrical features of the inkjet-printed nanosilver material for flexible electronics. Overview of the relevant literature published within last 5 years.

Nanosilver material	Ink composition	Printing system	Flexible substrate	No. of layers	Sintering method	Sheet resistance / Ω sq^{-1}	Ref.
AgNPs	Custom 6.91 wt% dispersion in DI water with addition of TRITONX-405, glycerol and MY-3000 as wetting and leveling agents	Flexible electronics printed	Photographic paper	4	130 °C for 3h	2.71	[75]
AgNPs	Custom 15 wt% dispersion in DI water with addition of Dispersan-5040, BYK-028, Spredox D-260 and Surfynol 465 additives	HP DeskJet 1112, Hewlett-Packard	Photographic paper	7	120 °C for 1h	1.2	[62]
AgNPs	Custom 15 wt% dispersion in water/isopropanol with addition of NaOH	Epson R330 color inkjet printer	Photographic paper	30	180 °C for 80 min	0.16	[63]
AgNPs	Commercial 50 wt% dispersion in triethylene glycol monomethyl ether; particle size d50 = 85 nm, d90 = 120 nm (Sicrys 115-TM119, PV Nano Cell)	PIXDRO LP50 with a Fujifilm Dimatix printhead	Uncoated paper substrate	2	Photonic curing (PulseForge 1200)	< 1	[76]
AgNWs	Commercial dispersion in ethanol; d = 20 nm, l = 2-5 μm (IST-NW-S30-ST,	Microelectronic printer (Shanghai Mifang Electronic Technolo-	PET	14	60 °C for 10 min	34.0	[77]

	Haitai Naxin Technology)	gy Co.)					
AgNWs	Commercial dispersion in ethanol; *d* = 20 nm, *l* = 2-5 μm (IST-NW-S30-ST, Haitai Naxin Technology)	Microelectronic printer, Shanghai Mifang Electronic Technology Co.	Clean PET	14	80 °C for 10 min	13.0	[78]
AgNPs	Commercial 30 wt% dispersion in ethylene glycol monoethyl ether (Advanced Nano Product Co. Ltd., South Korea)	DMP-2831, Fujifilm Dimatix Inc., USA	PET	5	Near-infrared (NIR) (DTX Co., Ltd., South Korea)	1.38	[79]
AgNPs	Commercial 80 wt% dispersion in DEG (PS-004, PARU Co., Ltd, Korea). Different mixed ratio of PS-004/EtOH/EG were prepared and further optimized.	DMP-2831, Fujifilm Dimatix	PET		350 °C for 60 min	9.56	[80]
AgNPs	Commercial 30 wt% dispersion in ethylene glycol (Sigma Aldrich 798738)	Thetametrisis FR-DEPOSIT	Pretreated PI	1	Thermal	32.23	[81]
AgNPs	Commercial 30 wt% dispersion in triethylene glycol monoethyl ether (DGP-40LT-15C, ANP Co., Ltd.)	DMP-2831, Fujifilm Dimatix	PEN		IPL (PulseForge 3200, NovaCentrix)	1.5	[82]
Amphiphilic AgNPs	Custom 10 wt% dispersion in H_2O/EtOH/EG	Epson Stylus L800 printhead	Glossy paper<br>Coated PET	4<br>4<br>4	IPL (Xenon X-1100)	3.85<br>0.57<br>19.7	This work

Clean PI

AgNPs = silver nanoparticles; AgNWs = silver nanowires; DI = deionized water; DEG = dyethylene glycol; EtOH = ethanol; EG = ethylene glycol (; ***

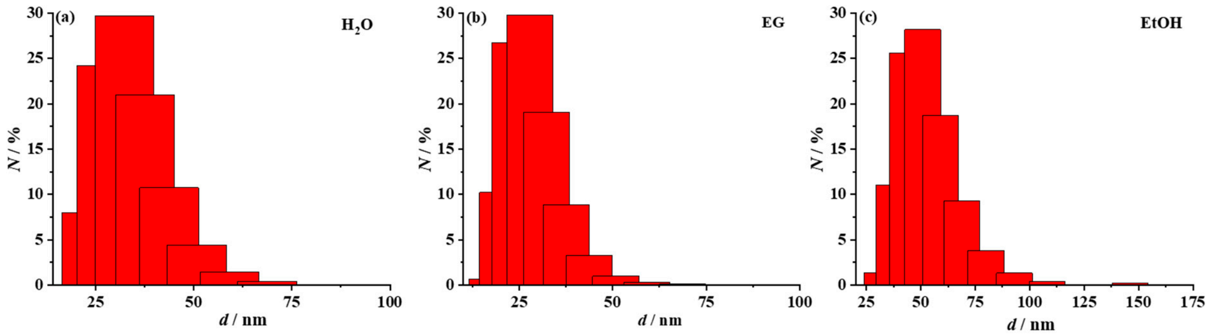

Figure S1. Histograms of PAA-capped AgNPs dispersed in **(a)** water; **(b)** ethylene glycol and **(c)** ethanol, respectively. Sample dilution of 1:200 by volume.

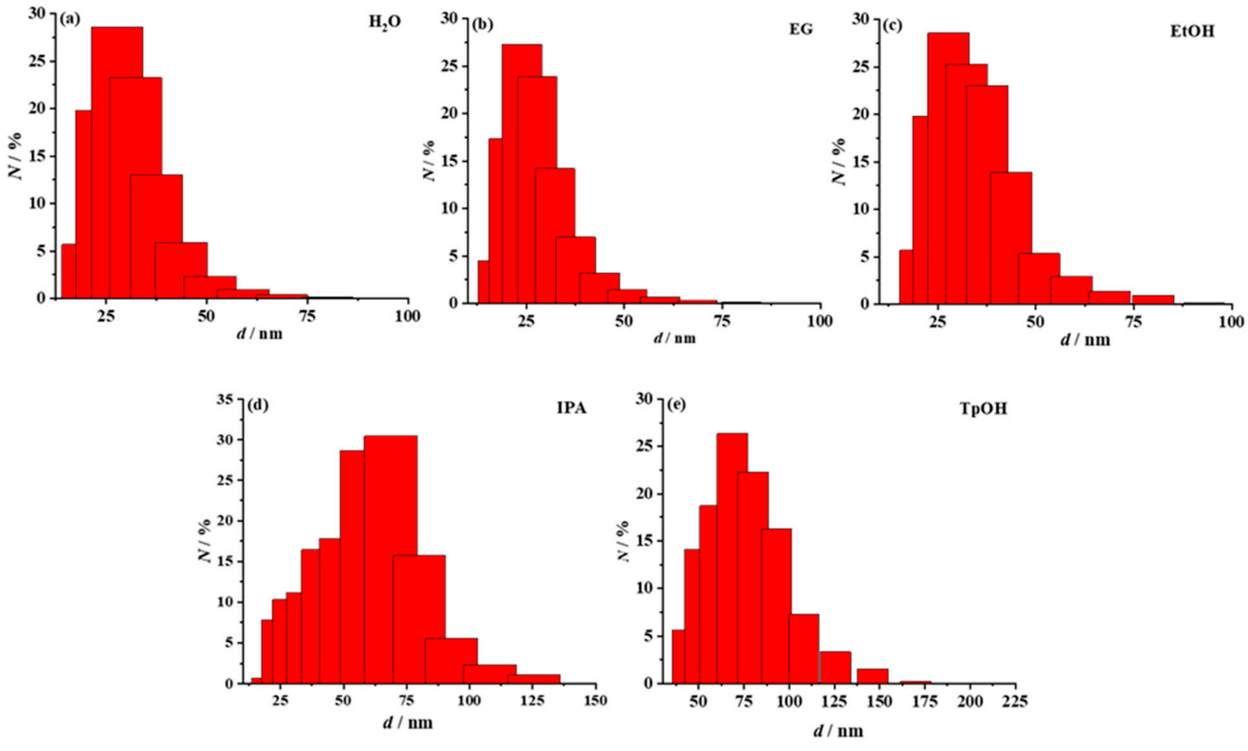

Figure S2. Particle size distribution of MPA-PAA-AgNPs, dispersed in pure solvents of different polarity: **(a)** H_2O; **(b)** EG; **(c)** EtOH; **(d)** IPA and **(e)** TpOH. Sample dilution of 1:200 by volume.

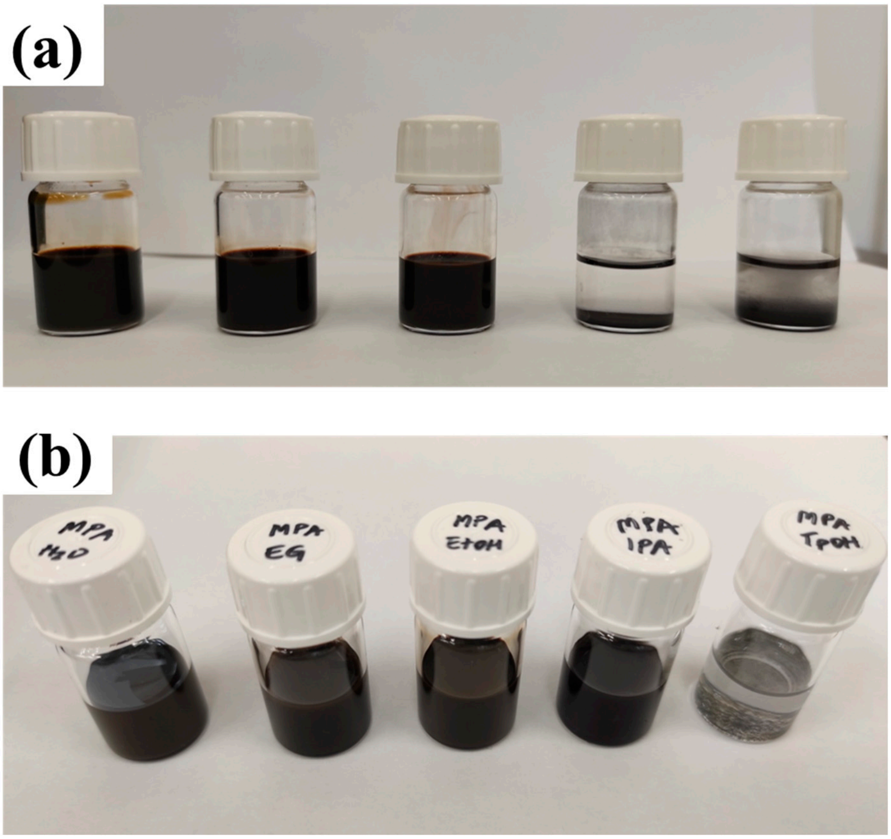

Figure S3. Suspensions of **(a)** PAA-AgNPs **(b)** MPA-PAA-AgNPs dispersed in pure solvents of different polarity, from left to right: water, EG, EtOH, IPA and TpOH, 30 days after preparation.

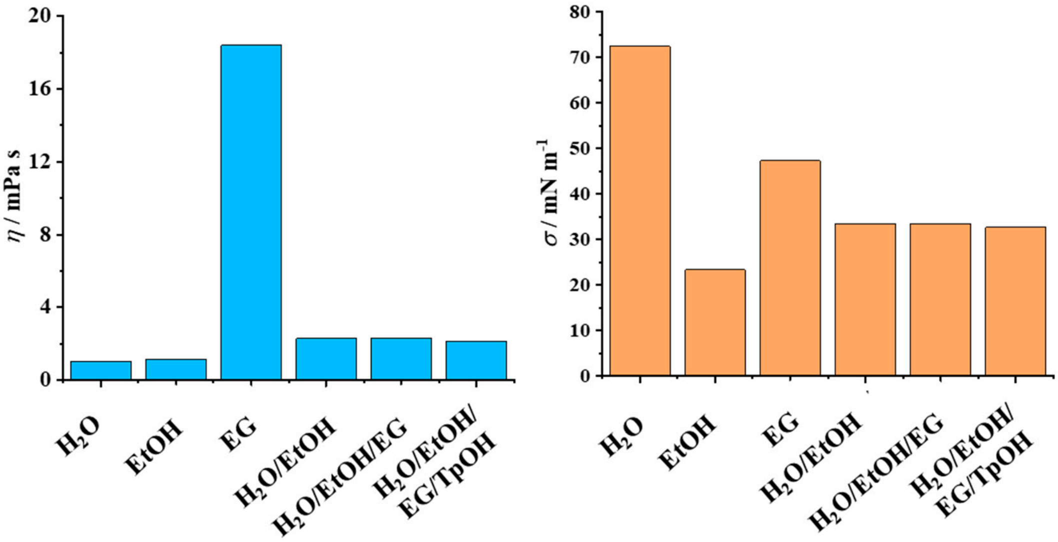

Figure S4. (a) Viscosity and **(b)** surface tension of pure solvents, binary, ternary and quaternary mixtures.

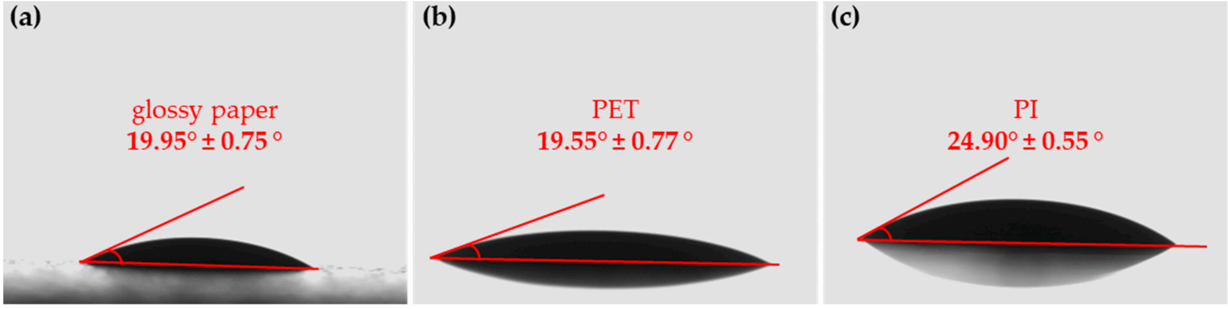

Figure S5. Surface wettability of the amphiphilic nanoparticles ink on the : (a) glossy paper; (b) PET and (c) PI surfaces, respectively.

References:

75. Wang, Y.H.; Du, D.X.; Xie, H.; Zhang, X.B.; Lin, K.W.; Wang, K.; Fu, E. Printability and electrical conductivity of silver nanoparticle-based conductive inks for inkjet printing. *J. Mater. Sci.-Mater. Electron.* **2021**, *32*, 496-508, doi:10.1007/s10854-020-04828-z.

62. Zhao, Y.Z.; Du, D.X.; Wang, Y.H. Preparation of silver nanoparticles and application in water-based conductive inks. *Int. J. Mod. Phys. B* **2019**, *33*, 1950385, doi:10.1142/s0217979219503855.

63. Hao, Y.Y.; Gao, J.; Xu, Z.S.; Zhang, N.; Luo, J.; Liu, X.Y. Preparation of silver nanoparticles with hyperbranched polymers as a stabilizer for inkjet printing of flexible circuits. *New J. Chem.* **2019**, *43*, 2797-2803, doi:10.1039/c8nj05639k.

76. Zikulnig, J.; Roshanghias, A.; Rauter, L.; Hirschl, C. Evaluation of the Sheet Resistance of Inkjet-Printed Ag-Layers on Flexible, Uncoated Paper Substrates Using Van-der-Pauw's Method. *Sensors* **2020**, *20*, 2398, doi:10.3390/s20082398.

77. Wu, X.L.; Wang, S.Y.; Luo, Z.W.; Lu, J.X.; Lin, K.W.; Xie, H.; Wang, Y.H.; Li, J.Z. Inkjet Printing of Flexible Transparent Conductive Films with Silver Nanowires Ink. *Nanomaterials* **2021**, *11*, 1571, doi:10.3390/nano11061571.

78. Wang, Y.H.; Wu, X.L.; Wang, K.; Lin, K.W.; Xie, H.; Zhang, X.B.; Li, J.Z. Novel Insights into Inkjet Printed Silver Nanowires Flexible Transparent Conductive Films. *Int. J. Mol. Sci.* **2021**, *22*, 7719, doi:10.3390/ijms22147719.

79. Sung, K.H.; Park, J.; Kang, H. Multi-Layer Inkjet Printing of Ag Nanoparticle Inks and Its Sintering with a Near-Infrared System. *Int. J. Prec. Eng. Manuf.* **2018**, *19*, 303-307, doi:10.1007/s12541-018-0037-8.

80. Zhou, L.; Chen, X.L.; Su, W.M.; Cui, Z.; Lai, W.Y. In-Depth Investigation of Inkjet-Printed Silver Electrodes over Large-Area: Ink Recipe, Flow, and Solidification. *Adv. Mater. Interfaces* **2022**, *9*, 2102548, doi:10.1002/admi.202102548.

81. Apostolakis, A.; Barmpakos, D.; Pilatis, A.; Patsis, G.; Pagonis, D.-N.; Belessi, V.; Kaltsas, G. Resistivity study of inkjet-printed structures and electrical interfacing on flexible substrates. *Micro and Nano Engineering* **2022**, *15*, 100129, doi:https://doi.org/10.1016/j.mne.2022.100129.

82. Mitra, D.; Mitra, K. Y.; Dzhagan, V.; Pillai, N.; Zahn, D. R. T.; Baumann, R. R. Work Function and Conductivity of Inkjet-Printed Silver Layers: Effects of Inks and Post-treatments. *J. Electron. Mater.* **2018**, 47, 2135-2142, doi:https://10.1007/s11664-017-6024-5
